# Novel Variations in the Celiac and Hepatobiliary Arterial Anatomy: A Cadaveric Case Report

**DOI:** 10.7759/cureus.60813

**Published:** 2024-05-21

**Authors:** Hosne Ara, Adegbenro O Fakoya

**Affiliations:** 1 Cellular Biology and Anatomy, Louisiana State University Health Sciences Center, Shreveport, USA

**Keywords:** anatomical variant, supraduodenal artery, accessory right gastric artery, celiac trunk, cystic artery

## Abstract

The celiac trunk and hepatobiliary anatomy often display variations in origin and branching patterns. A particularly rare variant involving the cystic artery giving rise to a common trunk for the supraduodenal and an aberrant right gastric artery, with an additional accessory right gastric artery originating from the splenic artery, has not been previously documented. We report a unique variation in the branching pattern of the celiac trunk and the cystic artery revealed during routine dissection of the hepatobiliary region of a male cadaver at Louisiana State University, Health Sciences Center, Shreveport. In this case, the cystic artery originated from the gastroduodenal artery and gave rise to a common trunk of an aberrant right gastric artery and the supraduodenal artery. Additionally, the cadaver lacked a proper hepatic artery, and an additional (accessory) right gastric artery originated from the splenic artery. This report is the first documented instance of such combined variations in the celiac and hepatobiliary arterial anatomy. Recognizing potential variations in these anatomies is crucial for radiological and surgical interventions in the hepatobiliary area to avoid iatrogenic hemorrhage or biliary complications.

## Introduction

In the surgical management of the hepatobiliary system, including procedures such as cholecystectomy, pancreaticoduodenectomy, liver transplantation, donor surgeries, and addressing traumatic injuries, it is crucial for surgeons to possess detailed knowledge of the topographic anatomy of the hepatobiliary arteries. Additionally, awareness of potential variations in these arteries is essential, as hepatic arterial anomalies may necessitate alternative surgical techniques. This knowledge is critical to prevent unintended hemorrhaging or biliary complications during these procedures [1].

The celiac trunk (CT), which supplies the hepatobiliary system, originates from the anterior wall of the abdominal aorta just beneath the aortic hiatus of the diaphragm, at the level of the intervertebral disk between T12 and L1 vertebrae [2,3]. Depending on the number of arteries arising from CT, bifurcation (CT branches into two arteries), trifurcation (CT branches into three arteries), quadrifurcation (CT branches into four arteries), and pentafurcation (CT branches into five arteries) pattern of the CT has been established [4,5]. It has also been reported that about 70-90% of cases of CT show its classical trifurcation branching pattern where CT divides into the left gastric, splenic, and common hepatic artery at the same point where it originates from the abdominal aorta [4,5].

After branching off from the CT, the left gastric and splenic arteries run, respectively, toward the lesser curvature of the stomach and the spleen. The common hepatic artery gives off the gastroduodenal artery, which travels inferiorly behind the duodenal bulb and further subdivides into the anterior and posterior superior pancreaticoduodenal artery and the right gastro-omental (gastro-epiploic) to supply the stomach, duodenum, and pancreas [3,6]. After giving the gastroduodenal artery, the common hepatic artery travels within the hepatoduodenal ligament and is referred to as the proper hepatic artery. Subsequently, the hepatic artery proper gives rise to the right gastric artery, which supplies the lesser curvature of the stomach and travels right toward the hilum of the liver, where it bifurcates into the right and left hepatic arteries to supply the liver [3,6]. The right hepatic artery then gives the origin of the cystic artery as a single stem from the right and posterior to the hepatic duct within Calot’s triangle and supplies the gallbladder [3,6]. It has been reported that apart from the proper hepatic artery, the right gastric artery also originates as a common trunk of the supraduodenal artery from the gastroduodenal artery [7].

Andall et al. conducted a review study about cystic arteries in over 9,800 cases. They concluded that 79.02% of cases of cystic arteries arise from the right hepatic artery, and the rest of the cases arise from other branches of the celiac arterial plexus. In 5.58% of cases, it arises from the aberrant right hepatic artery, in 2.07% of cases from the left hepatic artery, and in only 1.94% of cases, it originates from the gastroduodenal artery [8]. In 2021, Gish et al. published a case report where they reported that the cystic artery arises from the gastroduodenal artery as a common trunk of the cystic and supraduodenal arteries [9].

Although anatomical variations of the CT and its branches are common, this report highlights a seemingly undocumented variation in which the cystic artery arises from the gastroduodenal artery. It also gives the origin of the supraduodenal artery and the right gastric artery as a common trunk and an accessory right gastric artery arising from the splenic artery.

## Case presentation

**Figure 1:**
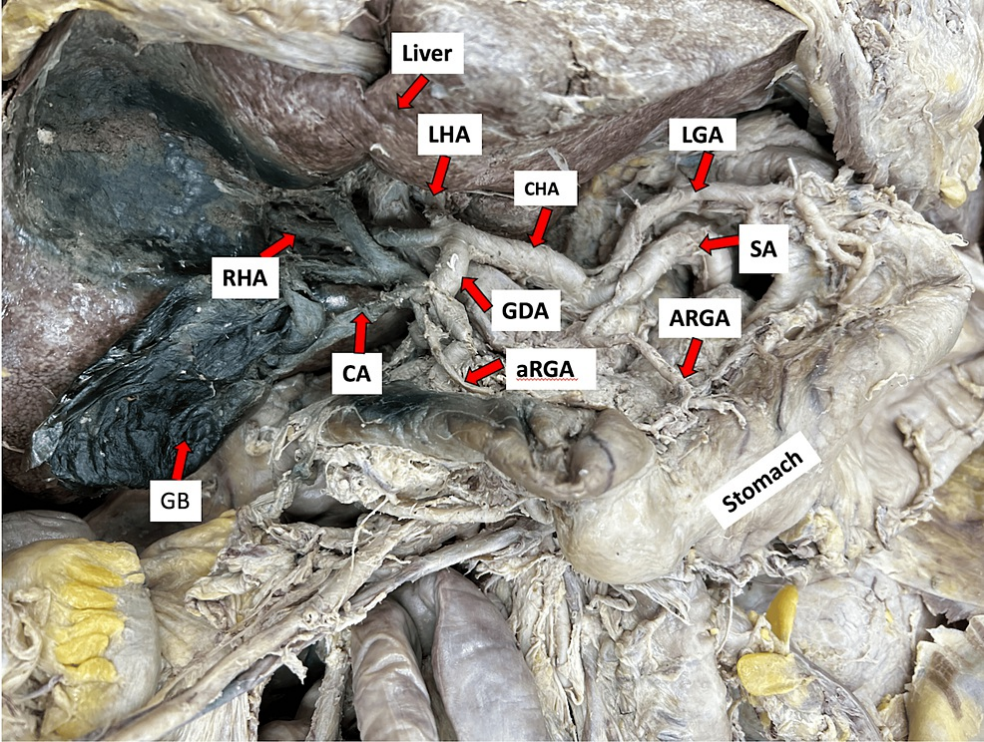
The dissection of the visceral surface of the liver showing variation of the celiac trunk and hepatobiliary anatomy. The celiac trunk gives rise to the left gastric, splenic, and common hepatic artery. The accessory right gastric artery originated from the splenic artery, and the cystic artery originated from the gastroduodenal artery. The cystic artery further gave off the supraduodenal artery and an aberrant right gastric artery. GB: gallbladder; CA: cystic artery; GDA: gastroduodenal artery; aRGA: aberrant right gastric artery; CHA: common hepatic artery; RHA: right hepatic artery; LHA: left hepatic artery; SA: splenic artery; ARGA: accessory right gastric artery; LGA: left gastric artery; HD: hepatic duct

This case report emerged from a routine dissection focusing on the liver and gallbladder area to investigate the arterial layout of the hepatobiliary region of an unnamed male cadaver at Louisiana State University, Health Sciences Center, Shreveport. During the dissection, we identified a trifurcated CT with a left gastric artery, splenic artery, and a common hepatic artery. The left gastric artery had the typical pattern, traveled retroperitoneally toward the esophageal hiatus, and descended along the lesser curvature to anastomose with the right gastric artery within the double-layered lesser omentum. Conversely, after branching off from the CT, the splenic artery atypically originated an artery that ran along the lesser curvature of the stomach, which we named the accessory right gastric artery supplying blood to the lesser curvature of the stomach. Following this, the splenic artery continued on its usual tortuous route along the superior and posterior margin of the pancreas toward the spleen. Subsequently, as we followed the path of the common hepatic artery and its branches toward the liver hilum, we noted a common hepatic artery that appeared normal and extended laterally to the right, giving rise to the gastroduodenal artery. However, immediately after this, the common hepatic artery bifurcated into the right and left hepatic arteries, as shown in Figure 1.

**Figure 2:**
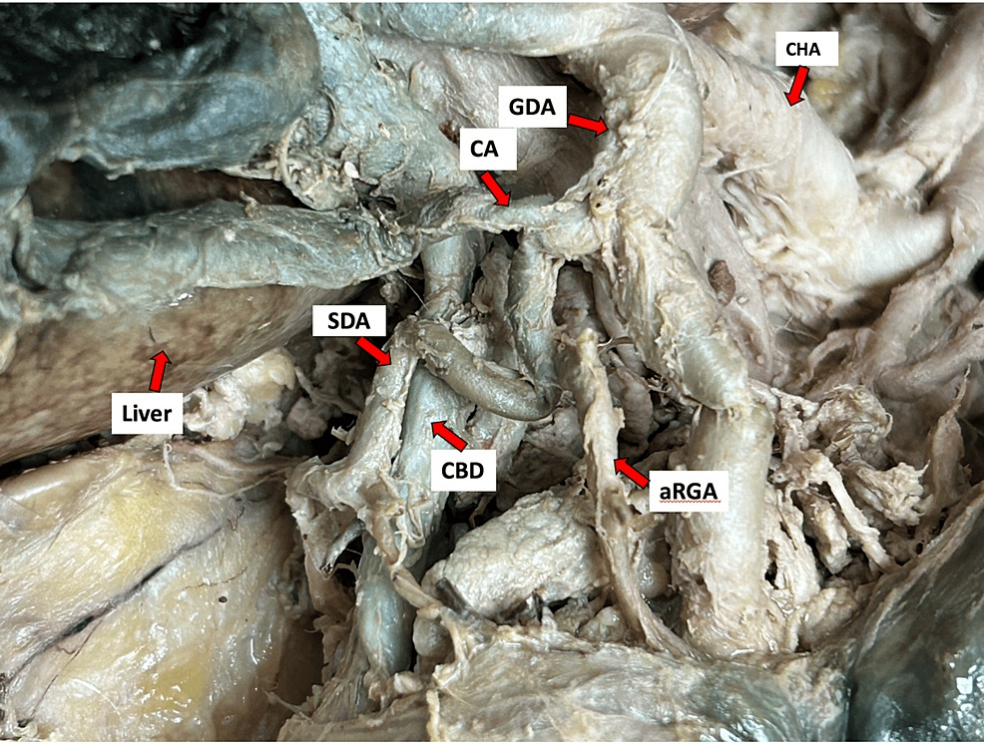
Close-up view of the celiac and hepatobiliary anatomy. This is a close-up view of the celiac trunk and hepatobiliary arterial anatomy showing the supraduodenal and right gastric arteries arising from the cystic artery. CA: cystic artery; GDA: gastroduodenal artery; aRGA: aberrant right gastric artery; CHA: common hepatic artery; aRGA: accessory right gastric artery; LGA: left gastric artery; SDA: supraduodenal artery

Typically, the common hepatic artery proceeds as the proper hepatic artery after branching off to form the gastroduodenal artery. Intriguingly, in this case, the common hepatic artery bifurcated directly into the right and left hepatic arteries, resulting in the absence of a proper hepatic artery in the donor. This anomaly prevented the typical identification of the cystic artery originating from the right hepatic artery. Upon further dissection, we unexpectedly discovered the cystic artery originating from the gastroduodenal artery, as shown in Figure 1 and Figure 2. Initially, the cystic artery gave off a common trunk for the supraduodenal artery and an aberrant right gastric artery; it then traveled along the anterior surface of the common bile duct toward the gallbladder, supplying it, as depicted in Figure 2. The supraduodenal artery was noted to extend toward the duodenum, while the aberrant right gastric artery headed to the lesser curvature of the stomach, each supplying their respective areas.

## Discussion

The literature extensively documents various anomalies in hepatic vasculature. Starting with the CT, numerous studies have shown variations in the origin and branching patterns of its subsequent branches. Typically, the CT splits into the common hepatic artery, splenic artery, and left gastric arteries from the abdominal aorta [7]. While an individual may possess a conventionally trifurcated CT, the branching patterns of its derivatives can often be atypical.

In the present case report, we observed multiple variations in the hepatobiliary region, including the origin of the cystic artery from the gastroduodenal artery with the subsequent origin of aberrant right gastric and supraduodenal arteries from the cystic artery, absence of the proper hepatic artery, and an accessory right gastric artery coming from the splenic artery. Understanding our findings may have clinical significance for abdominal vascular surgeries, liver transplantation, interventional angiography, and other abdominal procedures.

The cystic artery most commonly originates from the right hepatic artery within Calot’s triangle, which is delineated by the cystic duct, the common hepatic duct, and the inferior border of the liver [10]. Calot’s triangle is an important landmark for identifying the origin of the cystic artery. According to a previous report, the cystic artery exhibits frequent variation in the origin, position, and number of cystic arteries. In 2000, Chen et al. reported that cystic arteries also originate from the left hepatic, gastroduodenal, common hepatic, proper hepatic, superior mesenteric, supraduodenal, and superior pancreaticoduodenal arteries [11]. Recently, Gish et al. established that the cystic artery also originates from the gastroduodenal artery as a common trunk of the cystic artery and the supraduodenal artery [9].

In this case report, the cystic artery originated from the gastroduodenal artery as a single trunk. Despite this commonality, it uniquely gave rise to a common trunk for the aberrant right gastric and supraduodenal arteries, representing an undocumented and rare variation. The right gastric artery typically originates from the proper hepatic artery before it bifurcates into the right and left hepatic arteries. Then, it courses along the lesser curvature toward the left and anastomosis with the left gastric artery. Often, the right gastric artery originates from a source other than the proper hepatic artery within the celiac trunk. In such instances, these arteries are termed aberrant right gastric arteries, which are further classified as either replaced or accessory right gastric arteries [12]. Several groups reported other sources of the right gastric artery, which include the left hepatic artery (15-26%), the right hepatic artery (5-6%), and the middle hepatic artery (1.3-6%) [12]. Interestingly, in our donor cadaver, we observed a completely new variation where there was no hepatic artery proper, and an aberrant right gastric artery originated from the cystic artery. In addition, an artery originated from the splenic artery running along the lesser curvature of the stomach, anastomosing with the aberrant right gastric and left gastric arteries, which we named the accessory right gastric artery.

The use of laparoscopic and minimally invasive robotic-assisted surgeries is on the rise. During these procedures, inadvertent injury to blood vessels can necessitate conversion to open surgery [13]. Given the limited visibility of the patient’s anatomy in minimally invasive surgery, preoperative imaging becomes crucial for identifying anatomical structures and potential variations. It is particularly important for surgeons to be aware of our newly identified variation during laparoscopic, robotic-assisted, and traditional cholecystectomy procedures. One effective method to halt bleeding during surgery is to clamp or compress the injured vessel at its origin. Accidental ligation of the cystic artery before it branches into the right gastric and supraduodenal arteries could disrupt the blood supply to the donor’s stomach and duodenum. However, as noted in our observations, the presence of an accessory right gastric artery originating from the splenic artery could partially compensate for the blood supply to the stomach and duodenum in the event of accidental injury to these arteries.

## Conclusions

In this case report, we identified a novel variant of the cystic artery, which originated from the gastroduodenal artery and gave origin to an aberrant right gastric artery and the supraduodenal arteries as a common trunk. This patient also had a variation of the right gastric artery with an accessory right gastric artery originating from the splenic artery. We believe that this knowledge will help surgeons during planning or performing any hepatopancreatobiliary surgeries to prevent unwanted vascular complications and decrease morbidities.
